# Interstitial Pneumonitis in a Patient with Chronic Myeloid Leukemia

**DOI:** 10.4274/Tjh.2012.0115

**Published:** 2013-12-05

**Authors:** Ahmet Emre Eşkazan, Ayşe Salihoğlu, Serdar Erturan, Teoman Soysal

**Affiliations:** 1 İstanbul University Cerrahpaşa Faculty of Medicine, Department of Internal Medicine, Division of Hematology, İstanbul, Turkey; 2 İstanbul University Cerrahpaşa Faculty of Medicine, Department of Chest Disease, İstanbul, Turkey

**Keywords:** CML, Imatinib, interstitial pneumonitis

A 43-year-old otherwise healthy male patient was admitted to our hematology clinic with leukocytosis in April 2008. Philadelphia-positive chronic phase chronic myeloid leukemia (CML) was the diagnosis. Imatinib mesylate (IM) at 400 mg/day was initiated in May 2008. He tolerated the therapy quite well; only a mild skin rash developed, which was easily controlled with antihistamines. He was admitted with dry cough and shortness of breath, unresponsive to moxifloxacin therapy, in February 2010, after 20 months of IM treatment. He had a 15-pack-year history of smoking, which he had quit 2 years ago. He was afebrile and there were crackles on auscultation in both lungs. His chest X-ray showed bilateral interstitial infiltrates (Image 1A), and thoracic computed tomography (CT) revealed patchy parenchymal consolidations in both apical lungs, bilateral superior segments of the lower lobes, and the posterior regions of both lungs (Image 1B). Pulmonary function tests showed a restrictive ventilatory defect. No elevations of the acute phase reactants were detected, and the IgE blood level was within the normal limits. Fiberoptic bronchoscopy revealed normal airways, and bronchoalveolar lavage showed no abnormalities with the transbronchial fine needle biopsy showing nonspecific interstitial inflammation. The diagnosis was nonspecific interstitial pneumonitis and the possible cause was thought to be IM. Imatinib was discontinued and methylprednisolone (80 mg/day) was initiated. Under the corticosteroid therapy, his symptoms and radiological findings were resolved. 

Imatinib is indicated in the first-line treatment of CML. It is generally well tolerated, but mild adverse events including edema, nausea, diarrhea, abdominal pain, muscle cramps, and skin rash can be seen. Pulmonary complications such as dyspnea and cough are usually due to the pulmonary edema and pleural effusion that are seen in approximately 7%-14% of the patients receiving IM [1,2]. Interstitial pneumonitis during imatinib therapy is a rare entity [3]. While receiving IM and between days 10 and 337 of treatment, interstitial pneumonitis cases are documented [1]. The pathogenesis of the interstitial pulmonary disease during IM includes the direct toxic effects of the inflammatory and immune system cells resulting in parenchymal lung injury and fibrosis [4]. Our patient had tolerated IM well until month 20 of treatment, when a serious pulmonary complication occurred. Interstitial pneumonitis can be reversible when diagnosed early and treated quickly; thus, pulmonary symptoms in patients receiving IM should be closely monitored and possible pulmonary toxicity should always be kept in mind. Informed consent was obtained.

## CONFLICT OF INTEREST STATEMENT

The authors of this paper have no conflicts of interest, including specific financial interests, relationships, and/ or affiliations relevant to the subject matter or materials included.

## Figures and Tables

**Figure 1:**
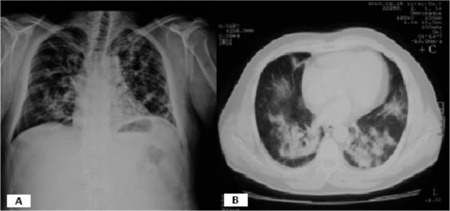
Chest X-ray showed bilateral interstitial infiltrates (A). Thoracic CT revealed patchy parenchymal consolidations in both apical lungs, bilateral superior segments of the lower lobes, and the posterior regions of both lungs (B).
